# Independent Component Extraction from the Incomplete Coordinate Time Series of Regional GNSS Networks

**DOI:** 10.3390/s21051569

**Published:** 2021-02-24

**Authors:** Tengfei Feng, Yunzhong Shen, Fengwei Wang

**Affiliations:** College of Surveying and Geo-Informatics, Tongji University, 1239 Siping Road, Shanghai 200092, China; 1911212@tongji.edu.cn (T.F.); wangfw-foster@tongji.edu.cn (F.W.)

**Keywords:** GNSS regional networks, ICA, independent component, data missing, signal reconstruction

## Abstract

Independent component analysis (ICA) is one of the most effective approaches in extracting independent signals from a global navigation satellite system (GNSS) regional station network. However, ICA requires the involved time series to be complete, thereby the missing data of incomplete time series should be interpolated beforehand. In this contribution, a modified ICA is proposed, by which the missing data are first recovered based on the reversible property between the original time series and decomposed principal components, then the complete time series are further processed with FastICA. To evaluate the performance of the modified ICA for extracting independent components, 24 regional GNSS network stations located in North China from 2011 to 2019 were selected. After the trend, annual and semiannual terms were removed from the GNSS time series, the first two independent components captured 17.42, 18.44 and 17.38% of the total energy for the North, East and Up coordinate components, more than those derived by the iterative ICA that accounted for 16.21%, 17.72% and 16.93%, respectively. Therefore, modified ICA can extract more independent signals than iterative ICA. Subsequently, selecting the 7 stations with less missing data from the network, we repeatedly process the time series after randomly deleting parts of the data and compute the root mean square error (RMSE) from the differences of reconstructed signals before and after deleting data. All RMSEs of modified ICA are smaller than those of iterative ICA, indicating that modified ICA can extract more exact signals than iterative ICA.

## 1. Introduction

In past decades, high-accuracy coordinate time series of global navigation satellite system (GNSS) stations have been widely used for monitoring seismic, coseismic displacements [1,2], and regional tectonic deformation [3]. The deformation signals, such as trend, annual and semiannual signals, as well as transit signals, can be detected from the GNSS time series, which contain abundant information from different sources, including tectonic and non-tectonic processes, such as the mass loading variations of snow, atmosphere and soil moisture [4,5,6,7]. The trend, annual and semiannual signals are usually estimated by the least-squares fitting. The other spatiotemporal signals are more effectively extracted and analyzed with some classic signal analysis methods, such as wavelet analysis (WA) [8,9,10], Kalman filter (KF) [11,12], empirical orthogonal function (EOF) [13], singular spectrum analysis (SSA) [14,15], and principal component analysis (PCA) [16,17,18,19]. Among these methods, PCA is one of the data-driven multivariate approaches based on second-order statistics (variance and covariance) and isolates the underlying sources without any prior knowledge [7], which implicitly assumes that a GNSS time series is polluted only by multivariate Gaussian noise. Nevertheless, previous studies demonstrated that a GNSS time series usually contains colored noise too [20,21]. Since PCA decomposition is based on the maximization of the variance of decomposed components, thus PCA works efficiently if only a single source exists in the GNSS time series. When multiple competing sources exist, however, the dominant components determined by PCA are probably the mixture from different physical sources [7,22].

In theory, GNSS coordinate time series can be regarded as a linear mixture of several independent signals from different physical sources, due to the unknown independent sources and mixing modes, the signal extraction is actually a blind source separation task [19]. Independent component analysis (ICA) approaches are the efficient statistical techniques for dealing with this kind of problem [23,24], which can separate the mixed signals into multiple statistically independent source signals based on high order statistics [25,26,27]. Recently, ICA has been successfully applied to analyze the geodetic data, especially the GNSS coordinate time series [5,6,22]. As in the PCA approach, ICA requires the processed time series to be complete. Data missing, however, inevitably occurs in GNSS coordinate time series, indicating that the interpolation should be implemented to fill the data gaps beforehand. Several commonly used interpolation methods in ICA, such as cubic spline interpolation [28], regularized EM interpolation [22], or iterative interpolation [29], may introduce false information and cause deviations in the extracted signals especially for the case of a large amount of missing data.

There are some theories for estimating the covariance matrix of incomplete data directly [30,31]. In addition, some improved PCA approaches have been developed, which need not fill the data gaps beforehand in dealing with incomplete time series [19,32]. In particular, the PCA approach was modified by Shen et al. [33] based on the principle that a time series can be reproduced with its principal components, this principle was further used to improve the singular spectrum analysis [34] and multi-channel singular spectrum analysis [35]. In this paper, the ICA is modified with two steps: first, the missing data are reconstructed using the improved PCA approach of Shen et al. [33], the independent components are then derived from the complete time series by using fast independent component analysis (FastICA) algorithm. In the following sections, the details of modified ICA theory is presented in Section 2, the preprocessing of experimental data is shown in Section 3, then the performance of modified ICA in extracting independent signals (except trend, annual and semiannual terms) is demonstrated in Section 4, and the conclusions are drawn in Section 5.

## 2. Methodology

### 2.1. Concept of Principal Component Analysis (PCA) and Independent Component Analysis (ICA)

If there are *n* stations of demeaned coordinate time series that span *m* epochs {x(t,j):t=1,2,⋯,m; j=1,2,⋯,n} (m≥n), these time series are stacked into an m×n matrix X=[x(t,1),x(t,2),⋯,x(t,n)], where the column vector x(t,j) denotes the time series of the *j*th station. In the PCA approach [16], the matrix ***X*** is decomposed as:

(1) $$X=PV^{T},$$

where, ***P*** is an m×n column orthogonal matrix and each column of ***P*** denotes a principal component (PC), the n×n eigenmatrix ***V*** is derived from the covariance matrix $B=X^{T}X$ with:

(2) $$B=V\Lambda V^{T},$$

where, Λ is an n×n diagonal matrix whose elements are sorted in descending order. Since ***V*** is an orthogonal matrix, the PC matrix can be derived as follows:

(3) P=XV

In detail, each row vector of ***P*** is derived from the corresponding row vector of ***X***. If the observation data are complete, Equations (2) and (3) can be directly used to solve the PC matrix. On the contrary, the above equations cannot be used for the incomplete observation data unless missing data are interpolated in advance. To avoid the limitation, according to Schoellhamer [36] and Shen et al. [33], when the missing data exist in the time series, the elements ***B***(*k*, *k*) and ***B***(*k*, *j*) of covariance matrix ***B*** are computed with:

(4) $$\left\{ \begin{array}{l} B\left(k,k\right)=\frac{1}{m_{k}-1}\sum_{t\in M_{k}}x\left(t,k\right)x\left(t,k\right)\\ B\left(k,j\right)=\frac{1}{m_{kj}-1}\sum_{t\in M_{k}\cap M_{j}}x\left(t,k\right)x\left(t,j\right) \end{array}\right.,$$

in which, $M_{k}$ and $M_{j}$ denote the data sets of station *k* and *j,* respectively. $m_{k}$ and $m_{j}$ denote the number of available epochs at station *k* and *j*, respectively. $m_{kj}$ denotes the number of epochs of the intersect set $M_{k}\cap M_{j}$. When there exist missing data at the *i*th row vector of ***X***, the correspondent row vector of the PC matrix ***P*** also cannot be directly computed with Equation (3). However, once the PC matrix ***P*** is available, the missing elements can be reproduced with Equation (1). Hence, when the missing data in Equation (3) are substituted with those from Equation (1), we can derive a rank-deficient system of equations to solve the row vectors of ***P***. Furthermore, to solve a rank-deficient equation, a minimum norm criterion should be introduced, for the details, one can be referred to Shen et al. [33]. The results demonstrated that this minimum norm solution outperforms the solutions with the interpolated missing data or the iterative solutions [33]. Once the matrix ***P*** is derived, the complete time series can be reconstructed with Equation (2).

Any pair of the derived PCs, i.e., the column vectors of matrix ***P***, are not mutually independent. To derive independent components, an n×n unitary matrix $W=\left[w_{1},w_{2},\cdots ,w_{n}\right]$ is introduced to rotate the PC matrix so that the column vectors after rotation are as independent as possible. Then Equation (1) is rewritten as:

(5) $$X=P\Lambda^{\;-1/2}WW^{T}\Lambda^{1/2}V^{T}=YA,$$

where, $Y=P\Lambda^{-1/2}W$, the column of Y represents the estimates of the independent components, $A=W^{T}\Lambda^{1/2}V^{T}$ called the mixing matrix. After $\Lambda^{-1/2}$ is introduced, the matrix $Z=P\Lambda^{-1/2}$ becomes a unitary orthogonal matrix, its column vectors are called normalized principal components. Once an appropriate W is given, the independent components are uniquely determined as Y=ZW. On contrary, the matrix W can be determined based on the condition that the column vectors of Y are as independent as possible. There are several methods to compute the matrix W, including the FastICA [24,25,26,27], joint approximate diagonalization of eigenmatrices algorithm [29,37,38], kernel independent component analysis [39,40], etc. In this work, the FastICA algorithm, which shows obvious advantages in computation, robustness and convergence rate [24], is used to estimate the rotation matrix W. It uses a fixed-point optimization scheme based on Newton iteration and an objective function related to negentropy, and the iterative solution $w_{k}$ is determined as follows [26,27]:

(6) $$\left\{ \begin{array}{l} w_{k}\left(l+1\right)=E\left[Z^{T}g\left(Zw_{k}\left(l\right)\right)\right]-E\left[g^{\prime }\left(Zw_{k}\left(l\right)\right)\right]w_{k}\left(l\right)\\ w_{k}\left(l+1\right)=w_{k}\left(l+1\right)/\left\|w_{k}\left(l+1\right)\right\| \end{array}\right.,$$

where *l* is the index of iteration, E is the operator of expectation, g(⋅) is the derivative of a kind of non-quadratic function, the second derivative $g^{\prime }\left(\cdot \right)$ is continuous and differentiable.

### 2.2. Modified ICA

PCA can be considered as a very useful strategy of decorrelation, which makes the separation procedure simpler and better conditioned [41]. As the modified ICA includes two processes, the first is to derive the minimum norm solution of PCs from the incomplete time series data and then calculate the complete unitary orthogonal matrix ***Z***, the second is to solve the rotation matrix W with the unitary orthogonal matrix ***Z*** by the FastICA algorithm. The independent component matrix is finally obtained as Y=ZW. We summarize the detailed algorithm flow in Algorithm 1.

In comparison, the main difference between modified ICA and ICA is that the way of solving the PC matrix ***P*** with the incomplete GNSS time series, in which the modified ICA can directly calculate the PCs, while ICA needs to be interpolated beforehand or iteratively computed. When there are no missing data, the two methods are equivalent. If the PC matrix is iteratively computed by Equations (1)–(3), where the missing data are filled with zero at the first step, then FastICA is used to determine the independent components, this approach is called iterative ICA in this paper.
**Algorithm 1** Modified ICA**INPUT:** GNSS coordinate time series with missing data.**OUTPUT:** Independent components. 1: Creating a matrix ***X***, which is consisted of *n* GNSS time series with missing data. 2: Constructing covariance matrix ***B*** with all available observations. 3: Deriving the principal component matrix ***P*** same as Shen et al. [33] 4: Calculating the unitary orthogonal matrix ***Z*** with $Z=P\Lambda^{-1/2}$. 5: **for** each rotation vector $w_{k}$
**do** 6:  Initialize $w_{k}\left(0\right)$. 7:  Set iteration counter *l* = 0 8:  $w_{k}\left(l+1\right)=E\left[Z^{T}g\left(Zw_{k}\left(l\right)\right)\right]-E\left[g^{\prime }\left(Zw_{k}\left(l\right)\right)\right]w_{k}\left(l\right)$ 9:  $w_{k}(l+1)=w_{k}(l+1)/\left\|w_{k}(l+1)\right\|$10:  **while**
$\left\|w_{k}\left(l+1\right)-w_{k}\left(l\right)\right\|>\varepsilon$
**do**11:    Increase counter l→l+1.12:    Updating $w_{k}$.13:  **end while**14:  Getting an independent component $y_{k}=Zw_{k}$.15:  Implementing the decorrelation for $w_{k+1}$ to avoid the same convergence direction16:  $w_{k+1}=w_{k+1}-\sum_{j=1}^{k}w_{k+1}^{T}w_{j}w_{j}$17: **end for**18: **return** all independent components ***Y***.%% ε is the pre-set threshold.

### 2.3. Significant Signal Extraction

The signal $S_{k}$ can be reconstructed with the corresponding independent component $y_{k}$ as follows,

(7) $$S_{k}=y_{k}a_{k},$$

where $S_{k}$ is an m×n matrix, $y_{k}$ is the *k*th independent component, $a_{k}$ is the *k*th row vector of the mixing matrix. $y_{k}$ and $a_{k}$ are respectively called the temporal response (TR) and spatial response (SR), which represent the common temporal varying function among different stations [6,22]. Then we compute the contribution ratio of *k*th independent signal with:

(8) $$r_{k}=\frac{\left\|S_{k}\right\|}{\sum_{k=1}^{n}\left\|y_{k}a_{k}\right\|}\times 100\;\%,$$

where ‖⋅‖ denotes the Frobenius norm, $r_{k}$ is the contribution ratio of the signal $S_{k}$. We rearrange all independent components in descending order according to their contribution ratios, thus the most significant signals of the time series can be represented by the first several distinctive independent components, which are expressed as:

(9) $$S=\sum_{k=1}^{d}y_{k}a_{k}$$

## 3. Real Data Analysis

### 3.1. Preprocessing of Experimental Data

**Figure 1:**
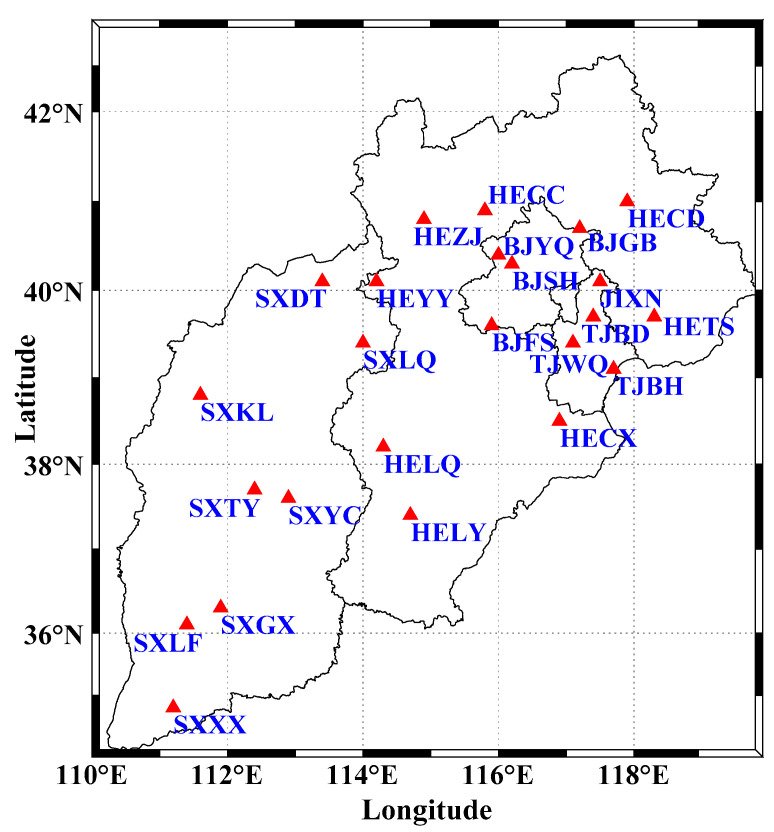
Distribution of 24 global navigation satellite system (GNSS) stations in North China.

**Figure 2:**
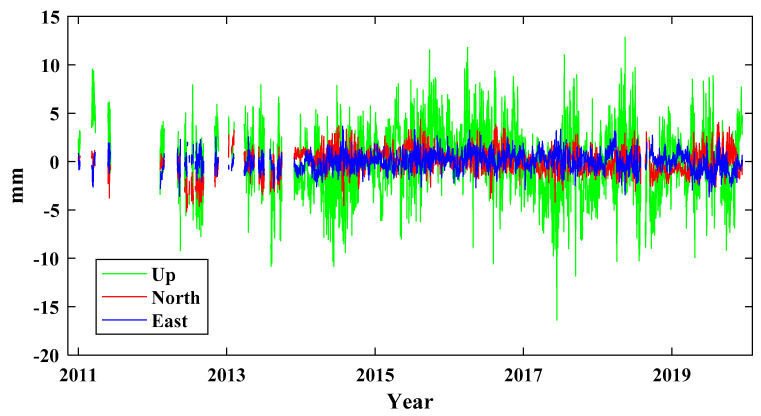
GNSS time series with missing data at station SXKL.

**Figure 3:**
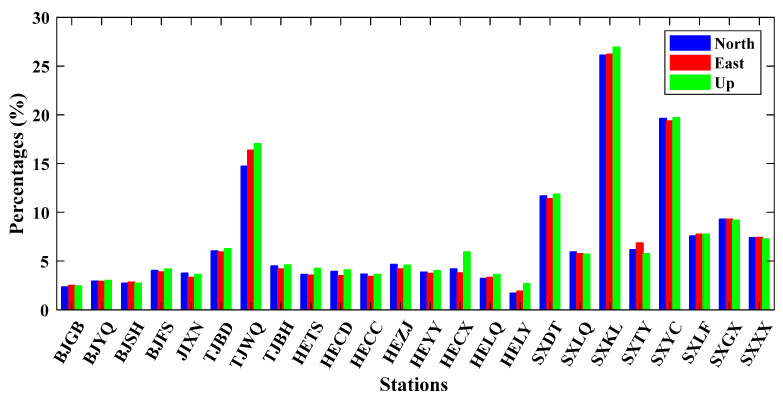
Missing percentages of 24 stations in North China from 2011 to 2019.

The coordinate time series of 24 GNSS stations in North China are collected from the Crustal Movement Observation Network of China (CMONOC) with a period from January 2011 to December 2019 (Figure 1) (available at http://www.cgps.ac.cn/ (accessed on 22 February 2020)). First, some unknown-type offsets are identified and corrected, and abnormal solutions with the formal errors larger than 50, 50 and 100 mm respectively for the North (N), East (E) and Up (U) coordinate components are detected and removed [16,22,28]. Then, a constant offset, trend, annual and semiannual terms are estimated and removed by the least-squares fitting from the original coordinate time series to derive the residual time series [4,42]. Moreover, when the absolute values of residuals exceed the pre-set thresholds of 10, 10 and 20 mm for the N, E and U coordinate components, they are also treated as outliers and removed further [22]. All the deleted outliers are regarded as a part of the original missing data. We show an incomplete time series at station BJFS in Figure 2 as an example. The missing data percentages of 24 stations are shown in Figure 3, and the means are 6.83, 6.82 and 7.13% for the N, E and U coordinate components, respectively. The correlation among the residual time series is first evaluated with the Kaiser–Meyer–Olkin (KMO) test [43,44], and the KMO values are 0.9186, 0.9461, 0.9603 for the N, E and U coordinate components, respectively. Therefore, strong correlations exist in the residual time series, which is suitable for the analysis of ICA.

### 3.2. Non-Gaussianity Assessment

**Figure 4:**
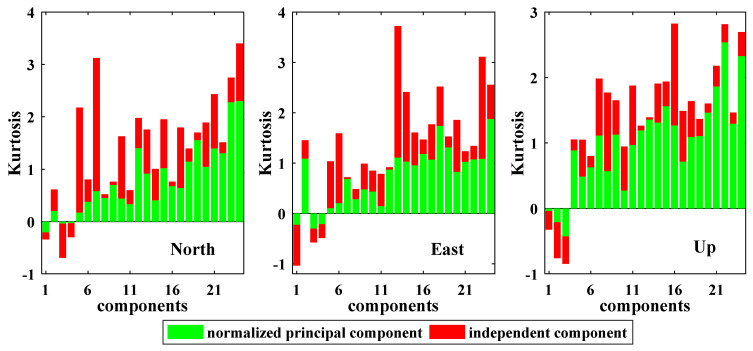
Map of kurtosis on the normalized principal components and independent components.

We tentatively explore the non-Gaussianity variations from normalized principal components to independent components. Following Forootan and Kusche [38], the non-Gaussianity of a component can be determined based on its kurtosis, which is used to measure whether data are peaked or flat relative to a normal distribution. Statistically speaking, kurtosis is more commonly defined as the fourth moment divided by the square of the second moment, i.e.,

(10) $$\mathrm{kurtosis}\left(\mu_{j}\right)={E\left[\mu^{4}\left(t,j\right)\right]\;/\;\left\{E\left[\mu^{2}\left(t,j\right)\right]\right\}}^{2}-3,$$

where Ε(⋅) is the expectation operator, $\mu_{j}$ represent the *j*th normalized principal component or *j*th independent component. If kurtosis = 0, the data have a Gaussian distribution, if kurtosis < 0, the data have a sub-Gaussian distribution, and if kurtosis > 0, the data have a super-Gaussian distribution. The kurtosises of 24 normalized principal components and 24 independent components derived from modified ICA are shown in Figure 4. It can be seen that the non-Gaussianities of the independent components are significantly stronger than those of the normalized principal components. According to the central limit theorem, the Gaussianity becomes stronger when more signals are mixed in a component. In other words, the stronger non-Gaussianity indicates that fewer signals are mixed. Therefore, it is proved that fewer signals are mixed in an independent component than the corresponding normalized principal component. The average absolute kurtosises of the independent components are 1.53, 1.35 and 1.43 for the N, E and U coordinate components, respectively. Therefore, the independent components possess a non-Gaussian distribution with high-order statistical properties.

### 3.3. Spatiotemporal Characteristics of the Independent Components

**Figure 5:**
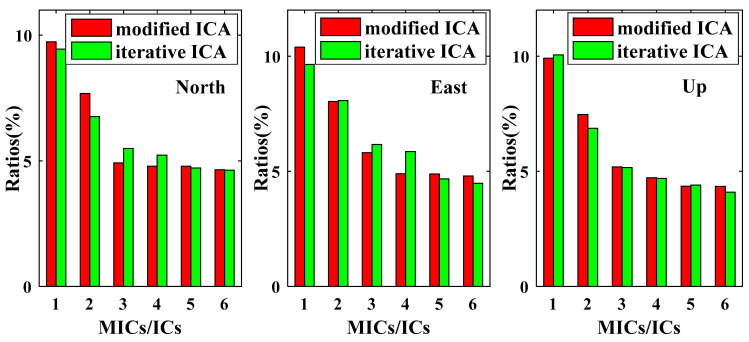
Contribution ratios of the first six modified independent component analysis (ICA)-derived independent components (MICs) and independent components (ICs).

The modified ICA-derived independent components (MICs) and the iterative ICA-derived independent components (ICs) are reordered according to their contribution ratios, and the histograms of the contribution ratios for the first six MICs and ICs are shown in Figure 5. The first MICs are larger than the first ICs for both N and E coordinate components, but slightly smaller for U coordinate component. The first two MICs account for the contribution ratios of 17.42%, 18.44% and 17.38% respectively for the N, E and U coordinate components, better than the first two ICs with the contribution ratios of 16.21%, 17.72% and 16.93%.

**Figure 6:**
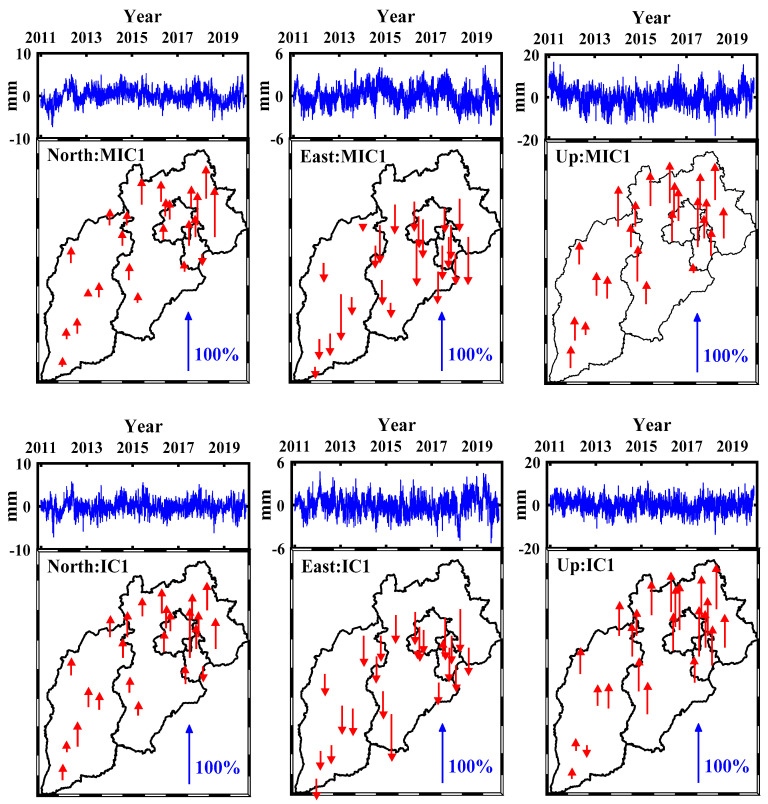
The first MICs and ICs for the three coordinate components. (**Top**) scaled temporal responses (TRs) and (**bottom**) normalized spatial responses (SRs).

**Figure 7:**
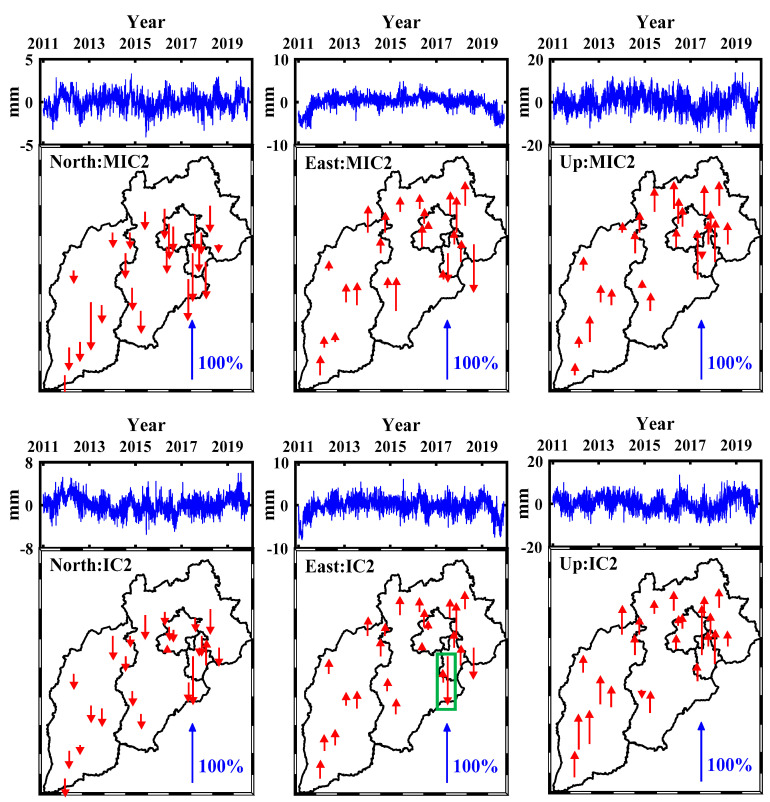
The second MICs and ICs for the three coordinate components. (**Top**) scaled TRs and (**bottom**) normalized SRs.

The SR and TR are usually used to demonstrate the spatiotemporal characteristics. Similar to Dong et al. [16], Each SR is normalized by dividing its maximum (absolute value) element, and corresponding TR is scaled by multiplying the normalization factor. All SR values, hence, are always in the range from −100% to 100%. The normalized SRs and corresponding scaled TRs of the first two MICs (MIC1 and MIC2) and ICs (IC1 and IC2) are shown in Figure 6 and Figure 7, in which the upward and downward arrows represent positive and negative SRs, respectively. The scaled TRs and normalized SRs derived from modified ICA are similar to those from iterative ICA for all three coordinate components, the mean values of the first two SRs (SR1 and SR2) derived from modified ICA are 49% and −31%, −54% and 34%, 45% and 37% for the N, E and U coordinate components respectively, while those from iterative ICA are 42% and −30%, −51% and 31%, 56% and 39% for the corresponding coordinate components. The SR1 values show a ladder-like distribution in the whole region, increasing gradually from south to north, and several large SR1 values are concentrated in the northeast of the studying area. By contrast, the notably different spatial patterns of SR2 can be seen, whose values show a more uniform pattern over the whole region. However, some stations show the opposite signs, e.g., TJBH in MIC1, IC1 and IC2 of N coordinate component, TJWQ in MIC2 and IC2 of E coordinate component. Moreover, some stations present significant difference compared to other stations, such as the TJWQ in IC2 of E coordinate component (in rectangle area of Figure 7), whose sign is negative and magnitude is much larger than any other station, which would bias the results to some extent [16], and thus we infer that the time series of TJWQ is polluted by the local effect [16,22].

Using the first two independent components to reconstruct the significant signals with Equation (9), and the Signal Power (SP) is then computed with the following expression,

(11) $$\mathrm{SP}=\sqrt{\frac{1}{n}\frac{1}{m_{j}}\sum_{j=1}^{n}\;\sum_{t\in M_{j}}S^{2}\left(t,j\right)},$$

where $M_{j}$ and $m_{j}$ denote the data set of station *j* and its number of available data points respectively, and *n* is the number of stations. Table 1 presents the SP values and overall time-consuming from modified ICA and iterative ICA, respectively. The SP values of modified ICA are 0.8177, 1.0254 and 2.7658 mm, respectively, for the N, E and U coordinate components, which are larger than 0.7904, 0.9583 and 2.6155 mm of iterative ICA for the corresponding coordinate components. The overall time-consumption of iterative ICA is longer than that of modified ICA, especially for the E coordinate component, which is due to the procedure of iterative interpolation which will affect the global efficiency to a certain extent.

## 4. Repeated Experiments Analysis

To compare the performances of modified ICA and iterative ICA in extracting independent components in the cases of different percentages of missing data, the following repeated data-deleting experiments were carried out. The seven stations with the least missing data, including BJGB, BJSH, BJYQ, HECC, JIXN, HEYY and HECD, were chosen for experiments. We used the first two MICs and ICs that were extracted from all available data of 7 stations to derive the reference signals for modified ICA and iterative ICA. The deleting percentages were from 5% to 30% with an increase of 5% each time, and the deleted data were randomly selected from the available data points of the 7 stations. After deleting different percentages of data, the first two independent components of the remaining data were used to reconstruct the signals. We repeated the experiments 200 times, the root mean squared error (RMSE) was adopted to evaluate the quality by examining the differences between reference signals and reconstructed signals.

(12) $${\mathrm{RMSE}}^{M}=\frac{1}{N}\sum_{k=1}^{N}\sqrt{\frac{1}{n}\frac{1}{m_{j}}\sum_{j=1}^{n}\;\sum_{t\in M_{j}}{\left(s_{k}^{M}\left(t,j\right)-s_{0}^{M}\left(t,j\right)\right)}^{2}}\;,$$

(13) $${\mathrm{RMSE}}^{F}=\frac{1}{N}\sum_{k=1}^{N}\sqrt{\frac{1}{n}\frac{1}{m_{j}}\sum_{j=1}^{n}\;\sum_{t\in M_{j}}{\left(s_{k}^{F}\left(t,j\right)-s_{0}^{F}\left(t,j\right)\right)}^{2}}\;,$$

where, the superscripts, ‘M’ and ‘F’, represent the values of the modified ICA and iterative ICA approaches, respectively. $s_{k}^{M}$ and $s_{k}^{F}$ are the reconstructed signals of modified ICA and iterative ICA respectively after deleting data, $s_{0}^{M}$ and $s_{0}^{F}$ are the reference signals. $M_{j}$ and $m_{j}$ denote the data set of station *j* and its number of data points involved in the experiment respectively. *n* is the number of stations and *N* is the number of repeated experiments. We further examined the RMSEs of the reconstructed signals at the deleting and non-deleting data points, and what should be noted is that in such a case, $M_{j}$ and $m_{j}$ denote the data set and its number of deleting or non-deleting data points at station *j*, respectively.

**Figure 8:**
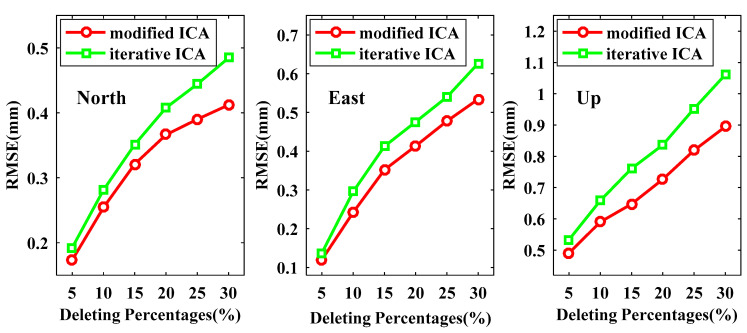
Root mean square errors (RMSEs) of the reconstructed signals derived from modified ICA and iterative ICA at all available data points.

**Figure 9:**
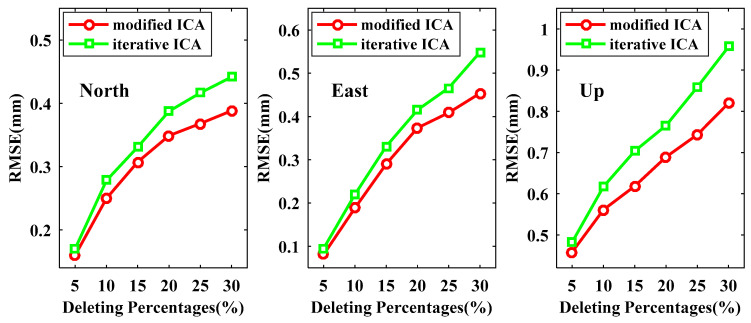
RMSEs of the reconstructed signals derived from modified ICA and iterative ICA at the non-deleting data points.

**Figure 10:**
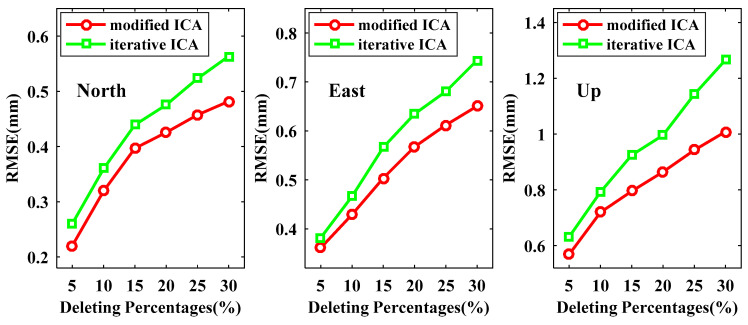
RMSEs of the reconstructed signals derived from modified ICA and iterative ICA at the deleting data points.

The RMSEs of reconstructed signals derived from modified ICA and iterative ICA at all available data points are presented in Figure 8, from which we can see that the RMSEs for both modified ICA and iterative ICA become larger when more data are deleted. All RMSEs of the reconstructed signals by our modified ICA are smaller than those by iterative ICA for the same experiment scenarios, especially for the U coordinate component. In the case of deleting 30% data, the improvements of modified ICA relative to iterative ICA are up to 14.96%, 14.75% and 15.67% for the N, E and U coordinate components, respectively. In Figure 9 and Figure 10, we demonstrate the RMSEs of reconstructed signals at non-deleting data points and deleting data points, respectively. Figure 9 shows that the RMSEs at the non-deleting data points vary similarly to the result of Figure 8. By deleting 30% of the data points, the RMSEs of modified ICA at non-deleting data points are 0.3882, 0.4583 and 0.8201 mm for the N, E and U coordinate components, while iterative ICA are 0.4415, 0.5487 and 0.9568 mm. Figure 10 shows that the RMSEs of reconstructed signals at the deleting data points are much larger than those at the non-deleting data points. When the data are deleted up to 30%, the RMSEs of modified ICA are 0.4819, 0.6507 and 1.0092 mm for the N, E and U coordinate components, respectively, which are smaller than the RMSEs of iterative ICA, i.e., 0.5636, 0.7442 and 1.2657 mm. Therefore, the proposed modified ICA extracts signal more exact residual time series compare to iterative ICA.

**Figure 11:**
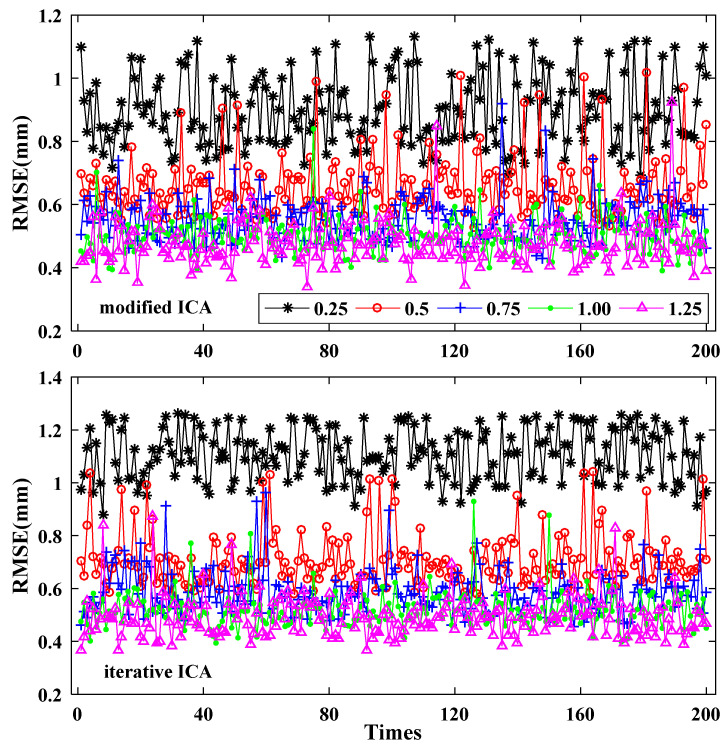
RMSEs of 200 experiments of modified ICA (**top**) and iterative ICA (**bottom**) in each signal-to-noise ratio (SNR) value (N coordinate component).

**Figure 12:**
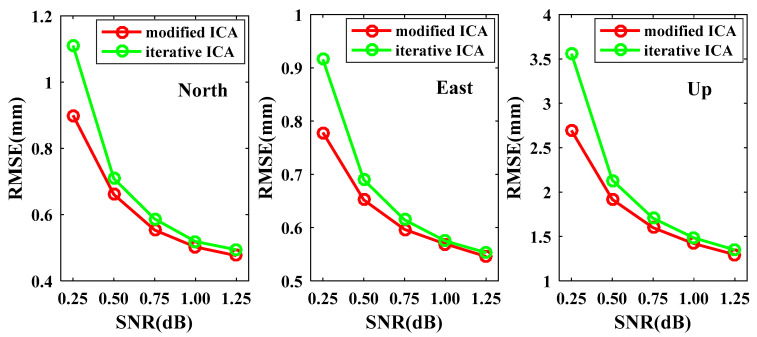
The mean RMSEs of modified ICA and iterative ICA for different SNRs.

Another repeated experiment was processed to further demonstrate the robustness and accuracy of the modified ICA method in extracting independent components under different noise levels. Similar to the above data-deleting experiments, the first two MICs and ICs that were extracted from the original time series of 7 stations were used to derive the complete time series of reference signals respectively for modified ICA and iterative ICA. Then, white noise and flicker noise were created according to the signal-to-noise ratio (SNR) values from 0.25 to 1.25 dB with an increment of 0.25 dB [22,28]. The new time series data were generated by adding the white and flicker noise to the reference signals and deleting the same epochs as the original time series. The experiments were also repeated 200 times. Since the reference signals were available for the new time series, the RMSEs of the reconstructed signals and reference signals were used to evaluate the quality of the two approaches. For the N coordinate component, the RMSEs of modified ICA and iterative ICA in each noise level are plotted in Figure 11, in which the results are very robust in 200 experiments. On the whole, the RMSE increases as the SNR value decreases for both modified ICA and iterative ICA, especially when the SNR value decreases from 0.5 to 0.25 dB. Figure 12 presents the mean RMSEs of modified ICA and iterative ICA for different SNR values. All the mean RMSEs of modified ICA are smaller than those of iterative ICA for the same SNR, which indicates that modified ICA can extract more exact signals than iterative ICA, especially when SNR is lower.

## 5. Conclusions

This paper developed a modified ICA approach for processing the incomplete time series of a regional GNSS network by using the reversible principle between the original time series and decomposed principal components. Twenty four GNSS residual time series of CMONOC in North China over the period 2011–2019 were processed to demonstrate the performance in extracting independent components with modified ICA and iterative ICA, respectively. The results showed that the contributions of the first two MICs accounted for 17.42%, 18.44% and 17.38%, respectively, for the N, E and U coordinate components, larger than the first two ICs that caught 16.21%, 17.72% and 16.93% of the total energy. The variations of TR1 and TR2 in the studying area were not significant, while the SR1 and SR2 showed different spatial patterns over the network. Subsequently, the first two MICs and ICs were used to reconstruct the significant signals, respectively, and the SP values of modified ICA were 0.8177, 1.0254 and 2.7658 mm for the N, E and U coordinate components, respectively, and 0.7904, 0.9583 and 2.6155 mm for iterative ICA, which indicated that modified ICA outperformed iterative ICA in extracting independent signals. Moreover, two repeated experiments were designed. The results of the first experiment with randomly deleting different percentages of total data showed that all RMSEs of modified ICA were smaller than those of iterative ICA. When the missing data accounted for 30%, the improvements of modified ICA with respect to iterative ICA were up to 14.96%, 14.75% and 15.67% for the N, E and U coordinate components, respectively. The second experiment by adding different noise indicated that modified ICA outperformed iterative ICA. Therefore, it is reasonable to conclude that modified ICA can indeed achieve independent components with higher accuracy than iterative ICA from the incomplete time series.

## Figures and Tables

**Table 1:** Signal power (SP) and time-consumption of modified ICA and iterative ICA.

Components	Modified ICA		Iterative ICA	
	SPs (mm)	Time (s)	SPs (mm)	Time (s)
North	0.8177	7.6	0.7904	7.8
East	1.0254	6.5	0.9583	15.6
Up	2.7658	6.2	2.6155	7.0

## Data Availability

Restrictions apply to the availability of the data used in this paper. Data were obtained from CMONOC and are available from http://www.cgps.ac.cn/ with the permission of CMONOC.
